# Behavior and thermal comfort of light and dark coat dairy cows in the Eastern Amazon

**DOI:** 10.3389/fvets.2022.1006093

**Published:** 2022-09-14

**Authors:** Welligton Conceição da Silva, Éder Bruno Rebelo da Silva, Maria Roseane Pereira dos Santos, Raimundo Nonato Colares Camargo Junior, Antônio Vinicius Corrêa Barbosa, Jamile Andréa Rodrigues da Silva, Juliana Amaral Vinhote, Eudilene Dalet Vitor de Sousa, José de Brito Lourenço Júnior

**Affiliations:** ^1^Institute of Animal Health and Production, Federal Rural University of the Amazon, Belem, Pará, Brazil; ^2^Institute of Agronomy, Federal Rural University of the Amazon, Capanema, Pará, Brazil; ^3^Institute of Engineering and Geosciences, Federal University of Western Pará, Belem, Pará, Brazil; ^4^Institute of Animal Science, Federal Institute of Education, Science and Technology of Pará, Santarém, Pará, Brazil; ^5^Cyberspace Institute, Federal Rural University of the Amazon, Belem, Pará, Brazil; ^6^Institute of Agronomy, Lutheran University Center of Santarém, Santarém, Pará, Brazil; ^7^Institute of Veterinary Medicine, Federal University of Pará, Belém, Pará, Brazil

**Keywords:** bioclimatology, stress, heat, ethology, Girolando

## Abstract

This study aimed to evaluate the behavior and thermal comfort of 20 Girolando cows (5/8-H/G), with light and dark coats, in the wettest period of the year, in Santarém, Pará, Brazil, in pasture with access to shade, and plenty of drinking water and mineral salt. Animal behavior categories were computed for 12 h a day, on 3 days in a row, by trained observers. Three day shifts were considered: Morning (6:00 a.m. to 9:55 a.m.), Intermediate (10:00 a.m. to 01:55 p.m.) and Afternoon (2:00 p.m. to 05:55 p.m.). The Temperature Index (TI), the Black Globe Humidity Index (BGHI) and the Comfort Index (CI) were calculated to measure thermal comfort. At all times studied, BGHI pointed that the environment was outside the thermal comfort zone. Dark-coated animals spent more 34.26% of the time in activities in the shade. The light-coated animals remained more 11.88% of the time in the sun, performing their natural behaviors. Both light and dark coat animals remained more 77 and 74.44% of the time in the sun, respectively. The behavior “in the sun while grazing” was the most evident, in both coats, in the studied shifts. The behaviors “in the shade while walking” and “in the shade while standing idle” were more evident (*p* < 0.01) in dark-coated cattle. The grazing behavior was higher in animals with dark coat (*p* < 0.05). In all evaluated shifts, there was a positive correlation between the behavior “in the sun while grazing” with the CI (r = 0.44211; *p* < 0.0305). Behaviors performed in the shade, such as “idleness while lying down,” “ruminating while lying down and standing up,” and behaviors “in the sun,” “idleness while lying down” and “ruminating while lying down,” were negatively correlated with CI. It is concluded that, even in the wettest period of the year, in the Eastern Amazon, Girolando dairy cows are exposed to hot environments, which causes thermal discomfort and changes in their natural behavior, as they spend more time standing in shaded areas, usually in rumination. Also, light-coated cows spend more time in the sun, while dark-coated cows spend more time in the shade. Thus, light-coated cows tend to have health and zootechnical performance negatively affected.

## Introduction

Throughout history, animals have been domesticated to meet the needs of humans, such as eating good quality meat (1–3). As a result of this approach, the behavior and lifestyle of these animals have also gone through changes, making it necessary to seek better strategies in order to increase production and productivity, taking into account their adaptations to environmental variables and appropriate management techniques. Thus, animal thermal comfort greatly contributes so as to the animal is able to express its full productive and reproductive potential (4, 5).

Relationship between animals and the environment is a key factor in the search for better productive efficiency in livestock, since there may be different responses from the animals, in relation to the characteristics of each region. Therefore, identifying variables that influence the animal's productive life, such as the stress caused by seasonal fluctuations in the environment, ensure suitable adjustments in production practices (6). The thermal comfort can be defined as a situation in which the thermal balance is zero (7). Therefore, heat stress is triggered when environmental conditions extrapolate the critical temperature range, consequently it is necessary to increase the basal metabolic rate for thermoregulation (8).

When heat stress is established in animals, different thermoregulatory mechanisms of animals are activated, depending a lot on external aspects such as coat color, length and hair type, promoting changes in behavioral and physiological responses, which affect productivity. Thus, animals under heat stress tend to increase respiratory rate and rectal temperature, reduce food intake, increase water intake and seek shaded areas to reduce thermal discomfort (9). In addition, these characteristics can be considered an evolutionary process or adaptation according to the environment (10).

In this context, many factors can cause negative effects on the physiological system of animals, such as high temperatures are associated with immunosuppression that can harm the health of animals, their feed efficiency in milk production systems or weight gain in animals. animals intended for meat (11–13). In addition, they can have implications for animal reproduction and fertility aspects such as hormone secretion or oocyte competence (14).

In addition, water limitation, shading, animal body temperature and behavior, when exposed to different air temperatures, directly affect the thermal heat exchange, in addition to latent heat losses (skin evaporation) to the environment, causing thermal stress in cattle (15). This situation is favored when there is no thermal balance between the animal and the environment, which can lead to serious productive and reproductive problems (16).

Therefore, it is deeply important to know the climate variables and their interactions with farm animals, in addition to their behavioral, productive and physiological responses in the adequacy of any production system, being necessary to combine measurement of animal values and environment, as a way to assess thermal comfort, aiming to establish measures to optimize environmental conditions (17), because when there is thermal stress, dairy cows tend to reduce milk production (18–21).

Coat color of cattle is considered a physical boundary established between environmental variables and the body of these individuals, influencing performance and production, as a result of its association with the animal's thermoregulatory mechanisms (22). Thus, coat color is an important factor, especially for cattle raised on pasture, in tropical climates, where the coat can influence the absorption of environmental heat (23–25).

Even though there are studies that point out the relationship between heat stress and animal production, it is noted that there is a gap regarding the influence of coat color, whether lighter or darker, on thermal comfort and behavior of dairy cattle, for example. in Girolando, as it is known that the amount of radiant heat absorbed is partially influenced by the coat color, thus, light-coated cattle tend to reflect more light than dark, which tend to absorb more (26). We hypothesized that light-coated cows spend more time in the sun and exhibit different behaviors than dark-coated cows during the rainy season in the eastern Amazon. Based on this information, this study aimed to evaluate the behavior and thermal comfort of Girolando cows, with light and dark coats, in the wettest period of the year, in Eastern Amazon.

## Materials and methods

### Local

**Figure 1:**
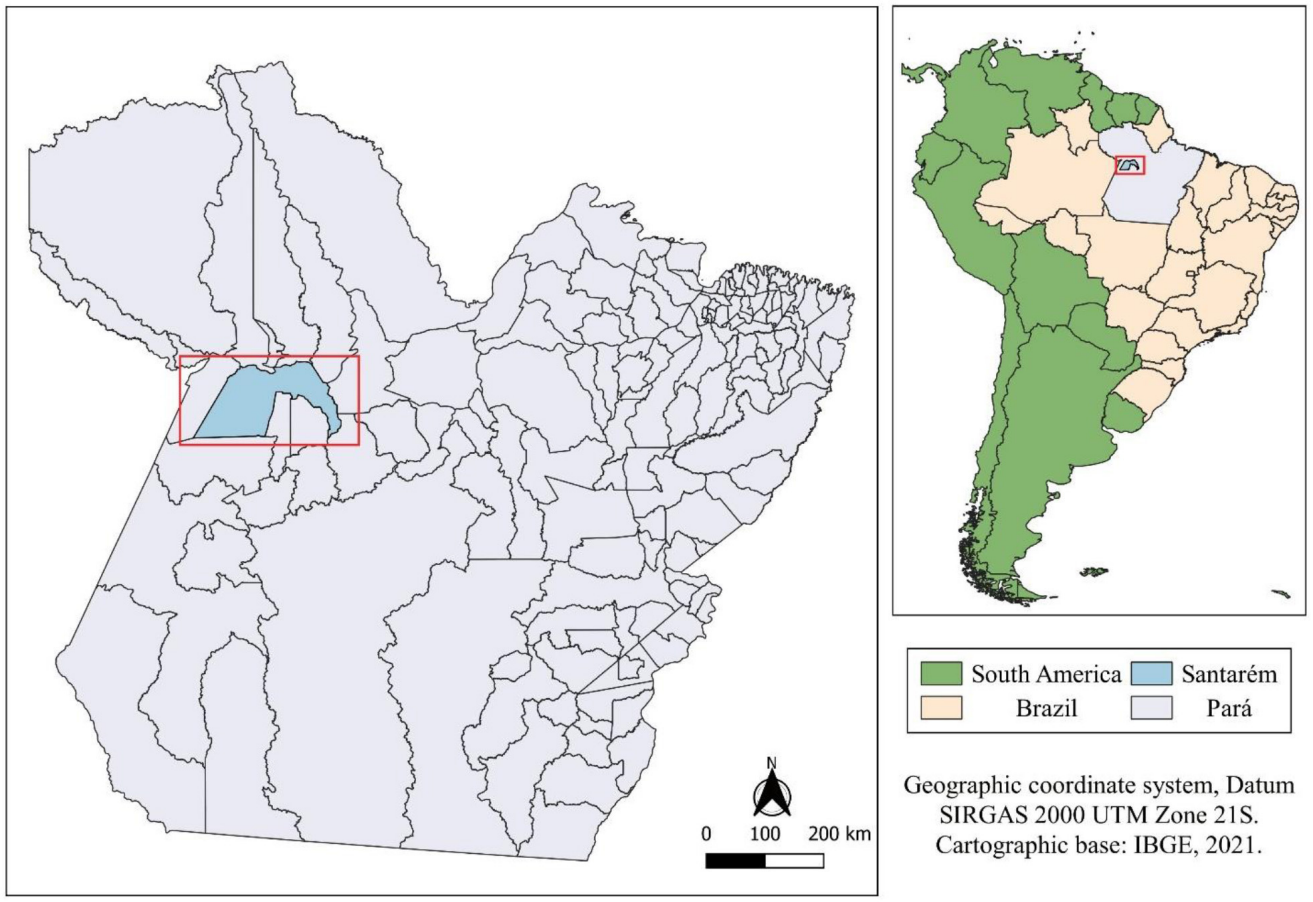
Location map of the studied area.

The study was carried out on a dairy farm, in Santarém, Pará, Brazil (Figure 1), in the wettest period of the year. The climate is hot and humid (Am4), as adapted by Köppen and Geise (27), with annual precipitation between 1,900 and 2,100 mm, average annual air temperature of 25.6°C and 82% relative humidity, ranging from 84 to 86%. The experiment was conducted in March 2021, which according to Martorano et al. (28), is within the wettest quarter of the year (February and April).

### Experimental animals

Twenty Girolando dairy cows, with a genetic ratio of 5/8 (H/G), average age of 22 ± 1.5 months, average weight of 312 ± 11.5 kg, non-pregnant. Cows were in mid-lactation (9 ± 2,52 liters/day; mean and SD). The animals were clinically healthy and were divided into two groups of light (white) (*n* = 10) (Treatment I) and dark (black) (*n* = 10) (Treatment II) coats. Cows were divided into two main groups with more than 85% black coat and more than 85% white coat (visual assessment) (18) subdividing to black hair samples and white hair samples. In this study, the animals had similar hair lengths. None of these cows showed cases of clinical diseases or disorders around calving.

It selected the darkest and lightest cows to participate in this study, excluding sick animals. Both groups remained in a single 3.5 ha paddock with *Brachiaria humidicola*, with access to shade, drinking water and mineral salt *ad libitum*. We emphasize that at the data collection site there were 25 trees with robust crowns (which would be equivalent to more than one tree per animal), preventing any social dominance between the light and dark groups of animals from impeding the bovines of both permanence groups in a place with shaded areas, this way the data becomes very reliable and expresses the current behavior of the animals.

### Ethogram

Behavior of the cows was observed for three consecutive days, from 7 am to 7 pm, totaling 12 h a day. The two groups of 10 animals were assessed every 5 min, according to the methodology adapted by Brscic et al. (29).

In the identification of the animals, visual observation was used, considering the phenotypic characteristics of each animal, making it possible to identify them individually. For this, four trained observers were used, divided into pairs, being replaced every 2 h, avoiding fatigue. Before the experiment, each observer was conditioned to evaluate and record the behavior of five equal animals for all observers, independently, and isolated in the field, for 5 h, aiming to evaluate the interobserver variation in relation to the behavior patterns. Interobserver variation was evaluated using the Kappa coefficient calculated in Microsoft Excel 2013 (Microsoft Corp., Redmond, WA), and an interobserver reliability of 90% was identified.

Behavioral variables were predefined, according to Coimbra et al. (30) and Agudelo et al. (31). Posture: two aspects considered, standing or lying down. Grazing, rumination and leisure activities. Frequency of water intake was observed, and the number of times the animal ingested water from the drinking fountain was noted. In addition to those described above, combination between the two behavioral variables was considered (Table 1).

**Table 1:** Ethogram: predefined behaviors according to the literature (30, 31) and their definitions.

**Behavior**	**Definition**
Standing	Leaning on its limbs, moving or stationary.
Lying down	Animal with four legs flexed and with the abdomen fully or partially in contact with the floor.
Grazing	The act of feeding on pasture, always standing.
Rumination	Animal chewing, swallowing, regurgitating and re-chewing with the presence of the apparent food bolus in the cheek space, which can be performed standing or lying down.
Idleness	Inattentive gaze in any direction with no apparent purpose, lying down or standing.
Walking	Animals moving without grazing inside the paddocks.

### Climate variables

Climate variables were checked, air temperature and relative humidity, obtained by means of a thermo-hygrometer (Brand: Incoterm; Model: 5203.03.0.00), being recorded every 15 min, from 7:00 am to 7:00 pm.

### Black globe temperature and humidity index

The BGHI was calculated, as proposed by Buffington et al. (32), using the equation (1). The values obtained indicate: ≤ 74: thermal comfort situation; 75–78: warning; 79–84: danger; and ≥85: emergency (33).


(1) $$\begin{array}{r} BGHI=TBG+0,36xTDP-41,5 \end{array}$$

Where: TBG is the black globe temperature (°C) and TDP is the dew point temperature (°C).

DPT was calculated using equation (2) proposed by Wilhelm (34).


(2) $$\begin{array}{r} DPT=\left(AT\left(-100-RH\right)/5\right) \end{array}$$

Where DPT is the dew point temperature (°C), AT is the air temperature (°C) and RH is the relative humidity (%).

### Thermal comfort index

Comfort Index (CI) was calculated in order to identify whether the thermal environment caused stress in dairy cows. The CI was determined by equation 3, adapted from Jones (34), considering the thermal discomfort zone for CI values >140.


(3) $$CI=T{(}^{{}^{\circ}}F)+RH(\%)$$

Where T is the air average temperature (°F) and RH is the air relative humidity (%).

### Statistical analysis

The experimental design was completely randomized (DCR). Statistical analysis was performed with non-parametric factorial DIC ANOVA using Artool library (aligned classification transformation). For a better understanding of the relationship between behaviors, a cluster analysis was performed, using the Ward. D2 minimum variation method, using the agricolae and factorextra packages, respectively, for behavior variables, as a function of the coat color of the animals (light and dark) as well as for shifts (morning, intermediate and afternoon). As data did not show normality, Spearman correlation was performed, considering 5% significance, in relation to the BGHI and CI, in order to signal positive and negative correlations that may influence the animals' activities. All analyzes were performed using software R version 3.4.1 (R Core Team 2016) (35).

## Results

**Figure 2:**
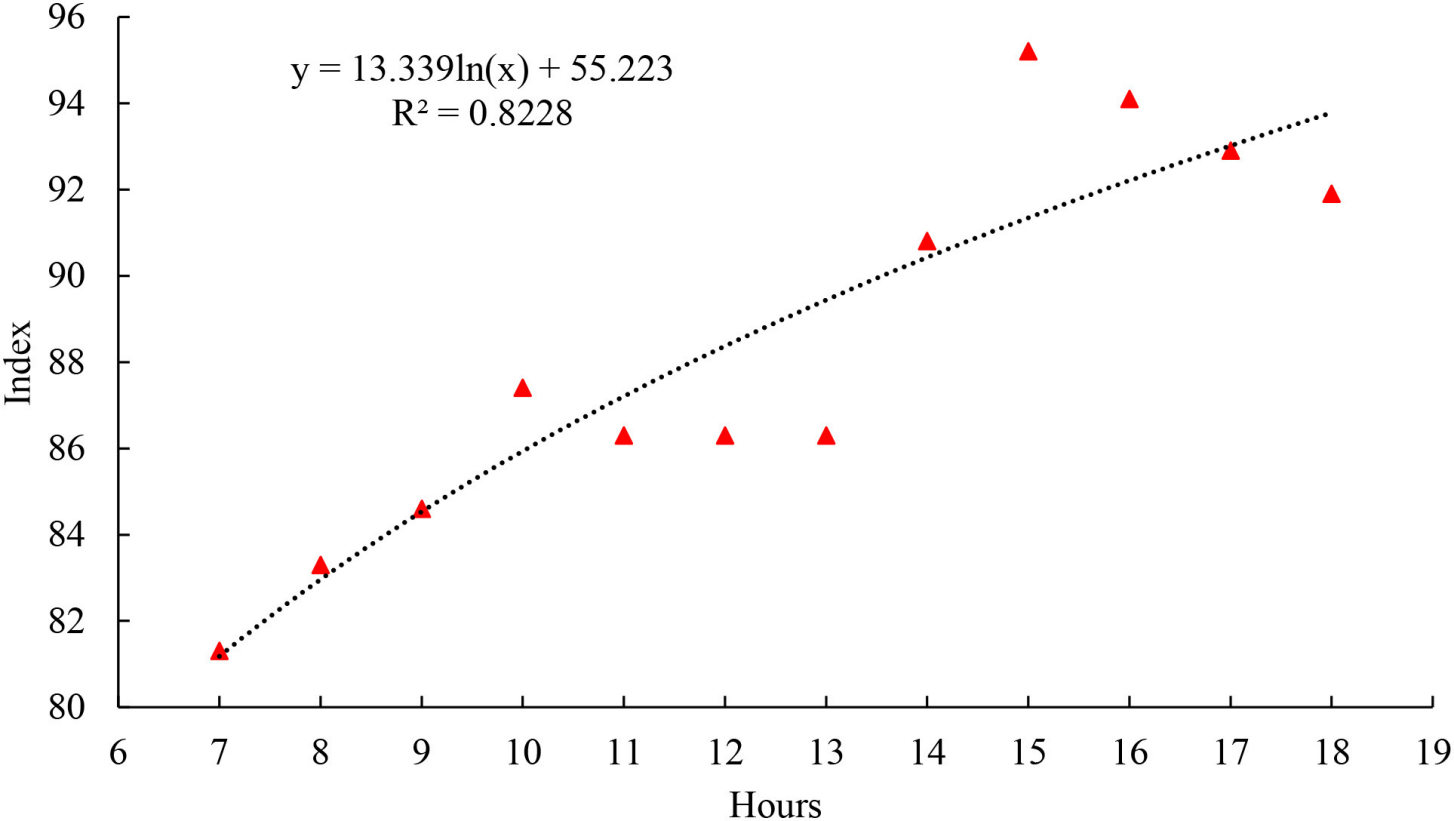
Regression of BGHI averages, according to the hours of the day.

There is an increase in BGHI over the hours of the day. All times signal BGHI outside comfort zone (74–78), and from 7:00 am to 9:00 am it was categorized as danger (79–84) and the others from 10:00 am to 6:00 pm, in a situation of emergency (≥85). In this scenario, cows ingested water more frequently between 3 p.m. and 5 p.m., that is, 32% of the ingestion took place at this time (Figure 2).

Table 2 shows data from descriptive statistics related to the behavior of animals with light and dark coats, in the intermediate, morning and afternoon shifts. The behavior “in the sun while grazing” (SH/G) was the most evident, both for light and dark-coated animals, in the studied shifts, with longer execution time of this behavior in the afternoon shift, in both coats, with values of 189.630 min in the light-coated cows and 175.926 min in the dark-coated ones.

**Table 2:** Descriptive statistics of behaviors related to light and dark color and shifts.

**Treatment**	**Shift**	**SH**						**SN**					
		**(minute)**						**(minute)**					
		**W**	**ILD**	**IS**	**G**	**RLD**	**RS**	**W**	**ILD**	**IS**	**G**	**RLD**	**RS**
Light	Intermediate	0.000	15.741	18.519	12.963	4.074	22.778	0.556	12.963	31.481	108.333	1.852	10.741
Light	Morning	0.000	0.556	8.519	13.148	0.000	1.296	1.111	0.556	51.111	162.593	0.000	1.111
Light	Afternoon	0.185	7.222	11.481	7.963	4.074	4.815	2.407	0.556	7.037	189.630	2.222	2.407
Total	-	0.185	23.519	38.519	34.074	8.148	28.889	4.074	14.075	89.629	460.556	4.074	14.259
Dark	Intermediate	4.259	16.111	45.926	36.296	12.963	13.889	16.111	3.519	11.667	78.333	0.926	0.000
Dark	Morning	0.926	1.296	17.963	12.778	1.667	1.481	15.000	0.370	13.889	174.630	0.000	0.000
Dark	Afternoon	0.000	4.074	14.074	13.148	3.889	1.852	9.815	4.444	6.667	175.926	0.556	5.556
Total	-	5.185	21.481	77.963	62.222	18.519	17.222	40.926	8.333	32.223	428.889	1.482	5.556

SH, shade; SN, Sun; W, walking; ILD, idleness while lying down; IS, idleness while standing; G, grazing; RLD, ruminating lying down; RS, ruminating standing.

It was evident that dark-coated animals spent 34.26% more time performing their activities in the shade, when compared to light-coated cows. Light-coated animals remained more 11.88% of the time in the sun, performing their behaviors, compared to dark-coated animals.

When analyzing the activities according to the treatment, it is noticed that both the animals with light and dark coats remained more 77 and 74.44% of the time in the sun, respectively, in different behaviors.

Behaviors such as “in the shade while walking,” “in the shade ruminating while lying down” and “in the sun ruminating while lying down” were not performed in the morning. But in the afternoon shift, these behaviors started to be performed by the light-coated cows.

In the case of animals with dark coat, the behaviors “in the sun while grazing” were more evident in all periods, with values of 78,333 min, in the intermediate period, 174,630 min in the morning and 175,926 in the afternoon. It is noteworthy that the behavior “in the sun ruminating while standing” was not observed in the intermediate period.

During the morning period, the behaviors “in the sun ruminating while standing” and “in the sun ruminating while lying down” were not observed. During the afternoon period, the behavior “in the shadow while walking” was not evidenced. There was a highly significant difference when comparing the animals with light and dark coats in activity “in the shade” and “in the sun” (*p* < 0.0001). There were differences between light and dark-coated animals between shifts (morning, afternoon and intermediate) (*p* < 0.0001).

**Figure 3:**
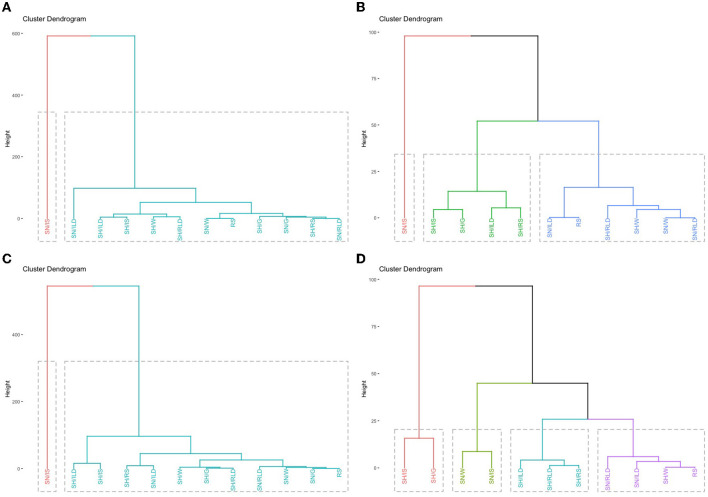
**(A)** Dendrogram of activities performed by animals. **(A)** Light colored animals with SN/G behavior. **(B)** Light colored animals without SN/G behavior. **(C)** Dark colored animals with SN/G behavior. **(D)** Dark colored animals without SN/G behavior. SH/W, shade while walking; SH/ILD, shade in idleness while lying down; SH/IS, shade in idleness while standing; SH/G, shade while grazing; SH/RLD, shade ruminating while lying down; SH/RS, shade ruminating while standing; SN/W, sun while walking; SN/ILD, sun in idleness while lying down; SN/IS, sun in idleness while standing; SN/G, sun while grazing; SN/RLD, sun ruminating while lying down; SN/RS, sun ruminating while standing.

In Figures 3A,B, it can be clearly seen, for both light-haired and dark-haired animals, that the behavior that stood out the most was SN/G (sun grazing), that is, grazing in the sun., forming a cluster separately on the left side of the figure, as, according to Table 2, it was the behavior with the longest time, 460,556 and 428,889 min, in light and dark, respectively, adding up the three shifts. The other behaviors, for the time they perform in each activity, were grouped into a single cluster, so to better visualize this second group formed, the SN/G behavior was excluded, which can be seen in Figures 3C,D. In this new cluster in the light coat (C), three groups can be observed, one only for the SN/IS behavior (sun in idleness while standing), a second group with the behaviors SH/IS (shadow in idleness while standing), SH/G (shadow while grazing), SH/ILD (shadow in idleness while lying down) and SH/RS (shadow ruminating while standing), and the third group with the other behaviors. In the dark coat (D), four groups, the first on the left with SH/IS behaviors (shadow in idleness while standing), with SH/G (shadow while grazing), a second group with SN/W (sun while walking) and SN/IS (sun in idleness while standing), a third group with SH/ILD (shadow in idleness while lying down), SH/RLD (shadow ruminating while lying down) and SH/RS (shadow ruminating while standing) and a fourth group with the other behaviors.

Regarding the behaviors performed by animals in the shade, there was a difference (*p* < 0.05) in the behavior “in the shade while walking” (SH/W), more evident in animals with dark coat (Table 3).

**Table 3:** Behavior of Girolando cows with light and dark coats, raised in the pasture, in the sun and in the shade, in the Eastern Amazon.

**Grazing in the shade**						
**Treatment**	**SH/W**	**SH/ILD**	**SH/IS**	**SH/S**	**SH/RLD**	**SH/RS**
Light	0.19^a^	23.52^a^	38.52^a^	34.07^a^	8.15^a^	28.89^a^
Dark	5.19^b^	21.48^a^	77.96^b^	62.22^b^	18.52^a^	17.22^b^
**Grazing in the sun**						
**Treatment**	**SN/W**	**SN/ILD**	**SN/IS**	**SN/S**	**SN/RLD**	**SN/RS**
Light	4.07^a^	14.08^a^	89.63^a^	460.56^a^	4.07^a^	14.26^a^
Dark	40.93^b^	8.33^b^	32.22^a^	428.89^a^	1.48^b^	5.56^b^

Averages followed by different lower case letters (a, b) in the columns are different (P < 0.05). SH/W, shade while walking; SH/ILD, shade in idleness while lying down; SH/IS, shade in idleness while standing; SH/G, shade while grazing; SH/RLD, shade ruminating while lying down; SH/RS, shade ruminating while standing; SN/W, sun while walking; SN/ILD, sun in idleness while lying down; SN/IS, sun in idleness while standing; SN/G, sun while grazing; SN/RLD, sun ruminating while lying down; SN/RS, sun ruminating while standing.

Regarding the behavior “in idleness while lying down,” there was no difference (*p* > 0.05) between animals with light and dark coats. However, there was a statistical difference (*p* < 0.05) between the behavior “in the shade in idleness while standing,” which was more evident in dark-coated cattle.

The grazing behavior was performed with greater intensity among dark-coated animals compared to light-colored ones (*p* < 0.05). On the other hand, the behavior “in the shade while ruminating lying down” showed no difference (*p* > 0.05), when associating animals with light and dark coats. The behavior “shadow ruminating while standing” showed a difference (*p* < 0.05) in animals of both coats, with this behavior being more performed by light-coated cows (28.89ª).

Also in Table 3 the behaviors performed by the animals exposed to the sun are described, in animals with light and dark coats. Differences (p <0.05) were identified in the behaviors “walking,” “idleness while lying down,” “chewing while lying down” and “ruminating while standing,” except for the behaviors “idleness while standing” and “grazing.” The light-coated animals performed the behaviors “idleness while lying down,” “chewing while lying down” and “chewing while standing,” more intensely than those with dark coat, which in turn performed the behavior “walking” more frequently.

Regarding the behavior of cattle in the shade for the intermediate, morning and afternoon shifts, in animals with light and dark coats, there was a difference (*p* < 0.05) in the behaviors “walking,” “idleness while standing,” “grazing” and “ruminating while standing” (Table 4), all of which are evidenced in the intermediate shift, with frequencies of 4.26th, 64.45th, 49.26th and 36.67th. In relation to the morning and afternoon shifts, there was no difference (*p* > 0.05) for the behaviors described above.

**Table 4:** Behavior of Girolando cows, raised in the pasture, in the sun and in the shade, at different times of the day, in the rainy season in the Eastern Amazon.

**Grazing in the shade**						
**Shift**	**SH/W**	**SH/ILD**	**SH/IS**	**SH/S**	**SH/RLD**	**SH/RS**
Intermediate	4.26^a^	31.85^a^	64.45^a^	49.26^a^	17.04^a^	36.67^a^
Morning	0.93^b^	1.85^b^	26.48^b^	25.93^b^	1.67^b^	2.78^b^
Afternoon	0.19^b^	11.30^c^	25.56^b^	21.11^b^	7.96^ab^	6.67^b^
**Grazing in the sun**						
**Shift**	**SN/W**	**SN/ILD**	**SN/IS**	**SN/S**	**SN/RLD**	**SN/RS**
Intermediate	16.67^a^	16.48^a^	43.15^a^	186.67^a^	2.78^a^	10.74^a^
Morning	16.11^a^	0.93^b^	65.00^a^	337.22^b^	0.00^b^	1.11^b^
Afternoon	12.22^a^	5.00^ab^	13.70^a^	365.56^b^	2.78^a^	7.96^a^

Averages followed by different lower case letters (a, b) in the columns are different (P < 0.05). SH/W, shade while walking; SH/ILD, shade in idleness while lying down; SH/IS, shade in idleness while standing; SH/G, shade while grazing; SH/RLD, shade ruminating while lying down; SH/RS, shade ruminating while standing; SN/W, sun while walking; SN/ILD, sun in idleness while lying down; SN/IS, sun in idleness while standing; SN/G, sun while grazing; SN/RLD, sun ruminating while lying down; SN/RS, sun ruminating while standing.

The behavior “ruminating while lying down” showed a (*p* < 0,05) difference between morning, afternoon and evening shifts, for light and dark-coated Girolando cows, in this case with the highest rate of execution of the behavior in the intermediate shift with 17.04^a^.

Table 4 shows the (*p* < 0.05) difference in the behaviors “in idleness while lying down” and “in the sun while grazing.” The activity “in idleness while lying down” was mainly performed in the intermediate shift, while the activity “in the sun grazing” was more evident in the morning and afternoon shifts. However, the behaviors “ruminating while lying down” and “ruminating while standing” were different (*p* < 0.05) in the intermediate and morning shifts, being more performed in the intermediate shift, with activity frequencies of 2.78a and 10.74a, respectively. On the other hand, when comparing the morning and afternoon shifts, no difference was spotted (*p* > 0.05), in cows with light and dark coats. In general, it was observed that the behaviors “walking” and “in idleness while standing” did not differ (*p* > 0.05) between shifts.

After Spearman's correction of the behavior data with the CI and the BGHI, it is noted that there was no correlation between the behaviors and the BGHI (*p* > 0.05). However, there is a correlation between behaviors and CI. There was a positive correlation between SN/G and CI (r = 0.44211; *p* < 0.0305).

**Figure 4:**
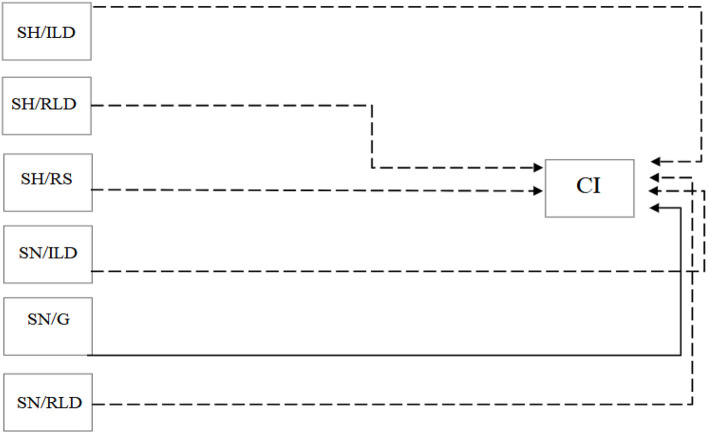
Positive (non-dashed lines) and negative (dashed lines) correlations between variables equally observed in all shifts and Black Globe temperature index and humidity (BGHI) and comfort index (CI). All correlations presented had *p* < 0.05. SH/W, shade while walking; SH/ILD, shade in idleness while lying down; SH/IS, shade in idleness while standing; SH/G, shade while grazing; SH/RLD, shade ruminating while lying down; SH/RS, shade ruminating while standing; SN/W, sun while walking; SN/ILD, sun in idleness while lying down; SN/IS, sun in idleness while standing; SN/G, sun while grazing; SN/RLD, sun ruminating while lying down; SN/RS, sun ruminating while standing.

Behaviors performed in the shade, such as “iddleness while lying down” (SH/ILD)(r = −0.42742; *p* < 0.0372), “ruminating while lying down” (SH/RLD)(r = −0.66782; *p* < 0.004) and “ruminating while standing” (SH/RS)(r = −0.68276; *p* < 0.0002) were negatively correlated with CI. The negative correlation with CI occurred in behaviors in the sun, such as “iddleness while lying down” (SN/ILD)(r = −0.49032; *p* < 0.0150) and “ruminating lying down” (SN/RLD)(r = −0, 48630; *p* < 0.0160) (Figure 4).

When the analysis was performed according to the treatment, a positive correlation was found between the behavior “in the sun in idleness while standing” (SN/IS) and the BGHI, and a negative correlation between the CI and the behaviors “in the shade ruminating while lying down” (SH/RLD), “in the shade ruminating while standing” (SH/RS) and “in the sun ruminating while standing” (SN/RS) (Table 5).

**Table 5:** Correlation between the behavior of Girolando cows, with light and dark coats and the Globe and Humidity Temperature Index (GHTI) and Comfort Index (CI).

**Light color animal**		
**Behavior**	**BGHI**	**CI**
SH_RLD	0.31412^a^ 0.3200^b^	−0.68743^a^ 0.0135^b^
SH_RS	0.12187^a^ 0.7059^b^	−0.75946^a^ 0.0042^b^
SN_IS	−0.64790^a^ 0.0227^b^	0.24869^a^ 0.4357^b^
SN_RS	0.28886^a^ 0.3625^b^	−0.68241^a^ 0.0145^b^
**Dark color animal**		
**Behavior**	**BGHI**	**CI**
SH_RLD	0.24875^a^ 0.4356^b^	−0.68591^a^ 0.0138^b^
SH_RS	0.07900^a^ 0.8072^b^	−0.62867^a^ 0.0285^b^
SN_ILD	0.10545^a^ 0.7443^b^	−0.70806^a^ 0.0100^b^

^a^Pearson's correlation coefficient. ^b^p-value. SH/RLD, shade ruminaring while lying down; SH/RS, shade ruminaring while standing; SN/ILD, sun in idleness while lying down; SN/IS, sun in idleness while standing; SN/RS, sun ruminaring while standing.

## Discussion

We observed that scientific data that consider the direct relationship between thermal comfort and coat color are still poorly explored (36). In this context, this study found that the light-coated girolando was more adapted to the tropical environments of the Eastern Amazon, even in the wettest season of the year, for animals raised in extensive systems, when compared to darker-coated animals.

When the thermal comfort zone is broken, different thermoregulatory mechanisms are activated, such as the increase in respiratory frequency and rectal temperature. In addition, animals tend to lose heat through cutaneous vasodilation or through conduction, convection and radiation as a result of the thermal gradient existing between the animal and the environment (9), thus, dark-colored cattle end up by absorbing more heat and presenting sharp drops in productivity.

Ambient temperature is considered one of the main factors for heat exchange, when this mechanism is not efficient and goes beyond the thermal comfort zone, thermoregulatory mechanisms are activated (37). Even in the wettest period of the year, the ITGU showed values of thermal discomfort in the animals. The presence of tree shade provided a reduction in DGHI indices, similar to what was observed by Magalhães et al. (38). However, even when there is no shading, the tree component is capable of altering the microclimate of the environment under the tree canopy (39–42). In this sense, the present work demonstrates that it is necessary to provide strategies to improve environmental conditions, because even in the wettest period, thermal stress can occur in animals, as observed in other studies (37, 40, 43, 44).

When animals are subjected to heat stress, there is a need for water to enhance heat exchange (45), this was verified in this study because there was a greater frequency of water intake in the afternoon, precisely when there was greater exposure to solar radiation, which provided greater evaporative losses (46).

Regarding coat color, dark-coated cows remained longer in the shade. This is because the morphological characteristics of the skin are intrinsically linked to thermal stress, as they have the ability to absorb about 93% of solar radiation. On the other hand, light-coated animals tolerated more sun exposure because this type of coat tends to reflect up to 60% more direct solar radiation when compared to dark-coated animals (24, 47–51).

These aspects were also observed in Holstein cows (29), in which the red coat phenotype showed a lower rate of absorption of solar radiation, therefore retaining less heat, when compared to the black color. In addition, black-coated Holstein cows showed greater cortisol retention and therefore higher levels of stress (52).

As discussed, it was found in this study that the light coat retained less heat, which ended up reflecting less heat stress when compared to the dark coat (53).

Regarding behavior, grazing was the most performed at all times of the day. This fact stems from the rhythmic circadian cycle of the animals, as the grazing activity tends to be performed with greater intensity, especially in the early hours of the day by cattle (53, 54).

Although cows are able to adapt to extreme temperatures, when subjected to these conditions for prolonged periods, they may have ruminal problems (55). For this reason, these animals try to maintain homeostasis by increasing food intake under low temperature conditions at different times of the day (56).

The present study indicated that there were different behaviors between shifts. This is because animals tend to distribute their activities such as grazing, ruminating and resting during the day (57, 58). Furthermore, animal behavior tends to change according to environmental characteristics (59).

It is noteworthy that heat stress can cause behavioral changes in order to reduce caloric production and/or promote heat dissipation, avoiding additional storage of body heat, which can reduce the grazing period and maximize the resting time of the animal cattle (60).

Rumination was the most evident behavior in environments protected from solar radiation, since shading helps to anticipate this activity, corollary to the better thermal comfort provided in this environment, as described by Deniz et al. (43), Pezzopane et al. (45), Sejian et al. (48), Titto et al. (61) and Giro et al. (62).

Some environmental conditions can directly affect this behavior, such as soil moisture, animal density, mud (63), in addition to rainfall and some pathological problems, such as lameness (64). For these reasons, cows perform activities such as grazing and walking in shaded environments, instead of resting, usually lying down (65).

The positive correlation between grazing and CI was due to the animals grazing at different times of the day, even in periods of heat and sun, in which the CI is outside the appropriate range. Thus, grazing is carried out standing, as a way of dissipating thermal energy through the convective path, which facilitates thermoregulation (66).

A negative correlation was observed between CI and SH/ILD, SH/RLD, SH/RS, SN/ILD and SN/RLD, because these activities were generally performed in the shade, regardless of the CI. However, when there was not enough shade available, the animals performed activities in the sun. On the other hand, the positive correlation between DPI and CI is due to the fact that the hottest hours of the day are used to practice this activity, usually in the shade.

The behavior in idleness, whether lying down or standing, was more evident in the intermediate period, that is, in the hottest part of the day, since cattle, when they are under heat stress, seek to minimize rumination time, aiming at thermal balance (67).

## Conclusions

Even in the rainiest period of the year, in the Eastern Amazon, Girolando dairy cows are exposed to hot environments, which causes thermal discomfort and changes in their natural behavior, as they spend more time standing in shaded areas, generally performing the rumination behavior. According to the present findings, it could be suggested that dark-coated cows have major thermoregulatory limitations when exposed to heat stress than light-coated cattle.

## Data availability statement

The raw data supporting the conclusions of this article will be made available by the authors, without undue reservation.

## Ethics statement

Ethical review and approval was not required for the animal study because as it is an observational study not involving the manipulation of animals to invasive processes.

## Author contributions

WS, ÉS, MS, JS, and JL: experiment design, experiment perform, and writing—original draft. WS, RC, AB, JV, and ES: data curation. WS and AB: formal analysis. All authors edited and approved the final manuscript.

## Funding

This study was funded in part by the Federal University of Pará and Coordenação de Aperfeiçoamento de Pessoal de Nível Superior (CAPES) Brasil (Finance Code 001) and also financial support for the publication fee from the Pró-Reitoria de Pesquisa e Pós-Graduação (PROPESP/UFPA).

## Conflict of interest

The authors declare that the research was conducted in the absence of any commercial or financial relationships that could be construed as a potential conflict of interest.

## Publisher's note

All claims expressed in this article are solely those of the authors and do not necessarily represent those of their affiliated organizations, or those of the publisher, the editors and the reviewers. Any product that may be evaluated in this article, or claim that may be made by its manufacturer, is not guaranteed or endorsed by the publisher.
